# The Association Between Sequence Learning on the Serial Reaction Time Task and Social Impairments in Autism

**DOI:** 10.1007/s10803-018-3529-6

**Published:** 2018-03-09

**Authors:** Fenny S. Zwart, Constance Th. W. M. Vissers, Joseph H. R. Maes

**Affiliations:** 1 Donders Institute for Brain Cognition and Behaviour, Radboud University, Montessorilaan 3, 6500 HE Nijmegen, The Netherlands; 2 Behavioural Science Institute, Nijmegen, The Netherlands; 3 Royal Dutch Kentalis, Sint-Michielsgestel, The Netherlands

**Keywords:** Implicit learning, SRT task, ASD, Social impairments, SRS-A

## Abstract

It is assumed that learning on the Serial Reaction Time (SRT) task is related to learning involved in social skill development affected in autism, but this assumption has hardly been investigated. We have therefore examined associations between SRT task learning and social impairment measured by the Social Responsiveness Scale in 72 autistic and non-autistic adults. Results revealed a positive correlation between deterministic sequence learning, putatively involving explicit learning, and social impairment in autistic adults but not in non-autistic adults. No correlations with probabilistic learning were found. These results suggest that the type of learning that helps autistic adults during a deterministic SRT task hinders them during social development, and call for further investigating the ecological validity of the SRT task.

**Electronic supplementary material:**

The online version of this article (10.1007/s10803-018-3529-6) contains supplementary material, which is available to authorized users.

## Introduction

Social communication skills are believed to develop largely through implicit, or automatic, learning mechanisms (Lieberman 2000). Learning what distance to keep or how to make small talk seems to come natural for most of us, without much explicit effort. This does not seem to be the case for people with Autism Spectrum Disorder (ASD), a neurodevelopmental disorder characterized by impairments in social communication skills (American Psychiatric Association 2013). This has led to the hypothesis that altered implicit learning mechanisms play a role in the development of ASD-related symptoms. Although some studies have found (subtle) learning problems or reported altered brain mechanisms during implicit learning in ASD (Gordon and Stark 2007; Mostofsky et al. 2000; Sharer et al. 2015, 2016; Travers et al. 2015; Zwart et al. 2017b), the majority of studies have found intact implicit learning in ASD (for meta-analyses see: Foti et al. 2015; Obeid et al. 2016; for a review see: Zwart et al. 2017a), hence challenging the hypothesized association between implicit learning as measured in scientific studies and social communication skills.

Most of these studies have used the Serial Reaction Time Task (SRT task; Nissen and Bullemer 1987). In this task, participants have to respond to a stimulus that appears on one of four locations on the screen as fast as possible by pressing a corresponding button. Unknown to the participant, these locations follow a sequence. Implicit learning is reflected by shorter reaction times (RTs) over time, without any (verbal) knowledge about the sequence. As a general reduction in RTs may reflect overall motor learning rather than sequence-specific learning, it is common to include (blocks of) random trials and investigate sequence learning as the difference in RTs between random and sequenced trials. The implicit nature of the task is confirmed by two features: (i) there is no intention to learn (i.e., no instruction), and (ii) there is limited awareness of the sequence knowledge. The latter is usually confirmed by post-experimental interviews, although other methods based on RTs are available too (e.g., Wessel et al. 2012). However, these measures show that a substantial number of participants do gain explicit knowledge in this task (e.g., Haider and Rose 2007). Such knowledge is believed to be prevented when random trials are inserted in the sequence, making the sequence probabilistic rather than deterministic (Jiménez et al. 1996; in the literature a probabilistic SRT task is often referred to as ‘Alternating SRT task’, see Howard and Howard 1997).

It is assumed that learning on the SRT task relates to the development of social communication skills (Lieberman 2000) and, hence, also that any deficits in learning on this task may be related to the social communication deficits in ASD (e.g., Mostofsky et al. 2000; Sharer et al. 2016). However, there is not much direct empirical evidence supporting these claims. One study found no correlation between ASD symptoms measured as raw scores on the Social Responsiveness Scale (SRS; Constantino and Gruber 2002) and implicit learning on the SRT task (Travers et al. 2010). In a later fMRI-study, the same researchers found that symptoms of repetitive behavior but not social communication deficits as measured by the Autism Diagnostic Interview-Revised (ADI-R; Lord et al. 1994) negatively predicted brain activation related to learning (Travers et al. 2015).

A factor complicating the interpretation of SRT task performance in ASD is the hypothesis that autistic participants use more explicit learning strategies (e.g., Klinger et al. 2007; Ullman and Pullman 2015). Such explicit strategies may lead to similar behavioral performance as implicit learning does, at least under certain conditions (e.g., Brown et al. 2010; Zwart et al. 2017b). It is therefore questionable whether we should interpret performance on the SRT task in ASD in terms of the same underlying learning mechanism (i.e., implicit) as we do for typical development (TD). Following this line of reasoning, an association between SRT task performance and social behavior impairments in ASD might reflect a relation between explicit learning and social functioning, whereas the same association in TD would reflect a relation between implicit learning and social functioning.

The aim of the current study was to further investigate the association between SRT task performance and social impairments and other autistic symptoms as measured by the questionnaire SRS for adults (SRS-A; Constantino and Gruber 2012). Given that social impairments measured on the SRS are continuously distributed amongst the general population (Constantino and Todd 2003), we first assessed this association in a group of 72 autistic and non-autistic individuals. As implicit learning is thought to play an important role in social skill development (Lieberman 2000), we predicted that overall, learning performance on a probabilistic and a deterministic condition of the SRT task would be negatively correlated to social impairments measured by the SRS. We expected this effect to be stronger during the (more implicit) probabilistic learning condition compared to the deterministic condition. Based on the hypothesis that autistic individuals may learn the task explicitly, whereas non-autistic individuals rely more on implicit learning, we also conducted within-group analyses to examine the possibility that the association between SRT task performance and social impairments may be different in autistic compared to non-autistic individuals.

## Methods

### Participants

Data from 72 participants from two studies was analyzed (see Table 1 for demographic details).

**Table 1:** Demographic details of TD and ASD participants from two studies

	Study 1		Study 2		
	TD group	ASD group	TD group	ASD group	*p*-value*
	(n = 19)	(n = 19)	(n = 18)	(n = 16)	
	*M* (*range*)	*M* (*range*)	*M* (*range*)	*M* (*range*)	
Age (years)	31.1 (20.6–57.1)	38.0 (18.5–59.8)			.11
			22.8 (18.8–29.7)	23.3 (19.8–27.6)	.57
Sex (F:M)	11:8	5:14			.10
			4:14	2:14	.66
IQ	109 (88–139)	111 (93–128)			.69
			114 (98–129)	107 (74–136)	.20
SRS-A	46.8 (36–60)	68.4 (51–88)			< .001*
			48.8 (36–65)	67.8 (49–94)	< .001*
AQ	12.3 (2–21)	29.2 (15–41)			< .001*
			12.9 (4–29)	26.3 (13–45)	< .001*

*Statistically significant *p*-values (< .005)

The first study included 19 autistic adults and 19 non-autistic adults from a previous EEG study (Zwart et al. 2017b). The second study included 16 young autistic adults and 18 young non-autistic adults. There was no overlap in participants between the two studies, i.e., none of the participants took part in both studies. All participants were free of major neurological disorders and all autistic participants were diagnosed with ASD by a clinician. Participants signed a written informed consent after being informed of the details of the study. Both studies were approved by a local ethical committee and in line with the guidelines of the Declaration of Helsinki.

### Procedure and SRT task

#### SRT task

**Fig. 1:**
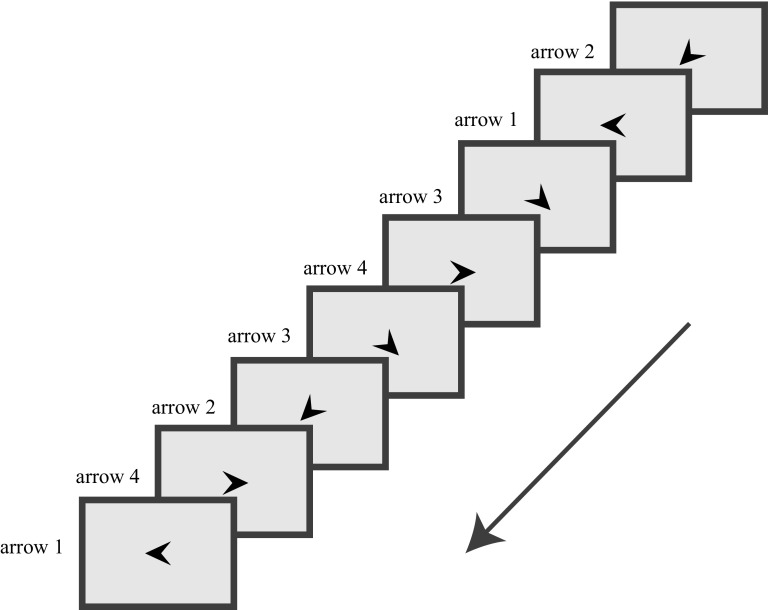
Serial reaction time task: the participant was asked to respond to the direction of the arrow, which—unknown to the participant—followed a repeating 8-element sequence (e.g., 2-1-3-4-3-2-4-1). Copyright by INSAR/Wiley Periodicals, Inc. (2017), adapted from Zwart et al. (2017b)

For study 1, the SRT task was administered while Electroencephalogram (EEG) was recorded for other purposes. The participant was instructed to respond to the direction of an arrow by a corresponding button press as fast as possible. Unknown to the participant, after 48 practice trials, the arrows followed a sequence (Fig. 1). We designed an SRT task that started with a probabilistic part, directly followed by a deterministic part. The probabilistic part of the task consisted of 72 repetitions of a probabilistic sequence (2-1-3-4-3-2-4-1) in which one stimulus in every sequence was replaced by a deviant (random) stimulus. In other words, the stimuli were only predictable with a certain probability. This part was directly followed by a deterministic part, consisting of 72 repetitions of a deterministic (i.e., no deviant stimuli) sequence (4-3-1-2-1-4-2-3). Both sequences were second-order in nature, in which two stimuli predicted the next stimulus (i.e., in the first sequence, ‘2-1’ predicted ‘3’). We ensured both sequences contained: (1) no repeating elements; (2) only one “serial” triplet (e.g., 1-2-3); and (3) only two “alternating” triplets (i.e., 1-2-1, 3-4-3). The deviant trials in the probabilistic sequence never repeated the adjacent sequenced trials, and equally represented the different stimuli. Response-to-stimulus interval was set at 500 ms. After the task, a short verbal interview was administered to assess levels of awareness of the final deterministic condition.

The SRT task used in study 2 was very similar to that used in study 1. The only three differences were: (i) only 60 (instead of 72) repetitions per sequence were used; (ii) deterministic sequence was slightly different (i.e., 4-3-4-1-3-2-1-2); (iii) within group, each participant received a different set of semi-randomized positions of the deviant trials. No EEG was recorded.

#### Other Measures

Participants were asked to fill out the SRS-A. The SRS-A consists of 65 items with a 4-point Likert-scale answer format and measures social impairments and related autistic symptoms (Constantino and Gruber 2012). Outcome of the SRS-A is a Total Score and four Subscale Scores: (1) Social Awareness, (2) Social Communication, (3) Social Motivation, and (4) Rigidity/Repetitive Behavior; with higher scores indicating higher levels of social impairments. It could be argued that the fourth subscale is not a direct aspect of social behavior, but rather indirectly related. Studies investigating the psychometric properties of the SRS have shown that reliability and validity are satisfactory (e.g., Bölte 2012; Chan et al. 2017; Gau et al. 2013). In addition, the participants filled out the Autism Quotient (Baron-Cohen et al. 2001), a questionnaire regarding autistic traits with 50 items that are answered with ‘definitely agree’, ‘slightly agree’, ‘slightly disagree’, ‘definitely disagree’, with total scores ranging between 0 and 50 (more details can be found in Baron-Cohen et al. 2001), and higher scores indicating higher degrees of autistic traits. The reliability and validity of the AQ have also been found to be satisfactory (e.g., Baron-Cohen et al. 2001; Hoekstra et al. 2008). An abbreviated version of the WAIS-IV (Wechsler 2008) was administered to estimate IQ, including the subtests Block Design, Similarities, Digit Span and Information. For 7 participants from study 1, a full WAIS-III (Wechsler 1997) had been administered within 12 months prior to participation, and this IQ-score was used instead.

### Statistical Analyses

For all main analyses, the alpha level was set at 0.05. Greenhouse Geisser correction was applied where the sphericity assumption was violated, and corrected statistics including adjusted degrees of freedom are reported. Effect sizes are expressed as partial eta squared (*ƞ*_*p*_^2^).

#### Data Preparation

For study 1, the RT data was split into 12 Blocks of 6 sequences (48 trials) in each condition to assess learning over time. For study 2, the RT data was split into 9 Blocks of 6 sequences. In order to make the two studies comparable, the last three blocks of study 1 were discarded from all analyses. Extreme outliers were determined by the Interquartile Range (IQR) criterion, i.e. values 1.5 × IQR ± the median RT over each Block for the standard trials, and over two large blocks for the deviant trials. On average, 24.2 (range 7–48; out of 378 trials; 6.40%) standard and 1.25 (range 0–5; out of 54 trials; 2.31%) deviant outlier trials were removed in the probabilistic condition, and 32.5 (range 8–60; out of 432 trials; 7.52%) outlier trials were removed from the deterministic condition. Trials with erroneous responses and the subsequent trials, as well as trials directly after a deviant trial, were removed.

#### ANOVAs of Probabilistic and Deterministic Learning

Although not the focus of the current paper, learning in the probabilistic condition was analyzed with a Group (ASD, TD) × Trial Type (Standard, Deviant) × Block (9) ANOVA, and in the deterministic condition with a Group (ASD,TD) × Block (9) ANOVA. Initial ANOVAs were conducted with Study (1, 2) added as additional between-subjects factor. Non-significant effects involving the Study factor confirmed comparable learning and justified pooling data from the two studies (see Supplementary Materials 1 for details of these analyses).

#### Relation Between Sequence Learning and SRS-A Scores

An overall probabilistic learning score was computed by subtracting the mean RT of all standard trials (Block 1–9) from the mean RT of all deviant trials (Block 1–9). An overall deterministic learning score was computed by subtracting the mean RT of the final Block (9) from the first Block (1). Pearson’s correlations between the probabilistic/deterministic learning score and SRS-A were analyzed. First, all participants (i.e., TD and ASD) were included to investigate social impairments as a spectrum. Because of the uncertainty regarding different learning mechanisms in ASD, subsequent within-group correlations were analyzed. To ensure that none of these correlations were due to outlier participants, data points with Cook’s distances > 1.00 were removed from the analysis (Cook and Weisberg 1982). Cook’s distances were determined by using a simple linear regression analysis with SRS-A score as independent variable, and learning score as dependent variable.

#### Differential Analyses on Age and IQ

In order to confirm that any potential correlational findings were not driven by the factors age and IQ, the same correlational analyses were conducted controlling for these factors.

## Results

### Block Analyses of Learning

**Fig. 2:**
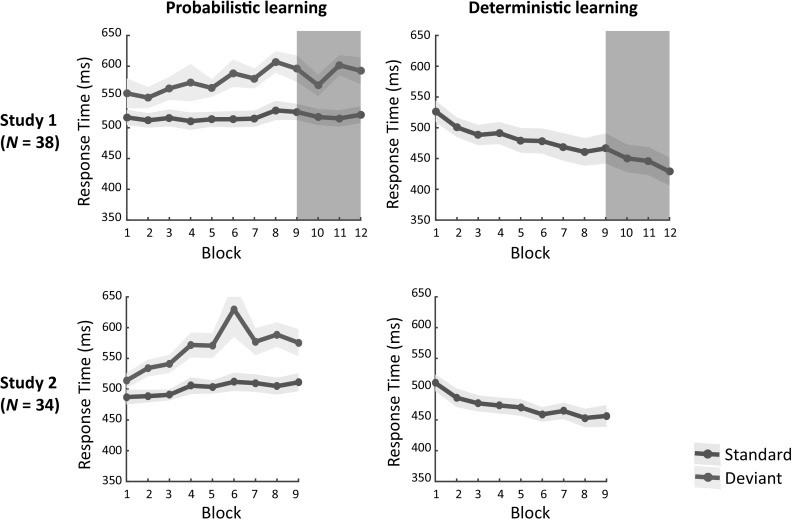
RT performance over blocks in the probabilistic condition (left) and the deterministic condition (right) for study 1 (upper) and study 2 (lower; shaded areas are standard error of the means; SEMs)

Figure 2 shows probabilistic and deterministic learning in both studies and suggests that these learning effects were similar. This suggestion was indeed statistically confirmed (see Supplementary Materials 1).

For the probabilistic condition, ANOVA revealed a main Trial Type effect, *F*(1,70) = 170, *p* < .001, *ƞ*_*p*_^2^=.71, with larger RTs for deviant (*M* = 571 ms) than standard trials (*M* = 510 ms), confirming sequence-specific learning. Furthermore, significant Block, *F*(4.7,328) = 4.8, *p* < .001, *ƞ*_*p*_^2^=.064, and Trial Type × Block interaction, *F*(4.3,299) = 3.8, *p* = .004, *ƞ*_*p*_^2^ = .052, reflecting a linear trend, *F*(1,70) = 17.5, *p* < .001, *ƞ*_*p*_^2^ = .20, effects suggested that learning increased over time. No effects involving the Group factor were found, *p*’s ≥ .19, suggesting similar motor speed and learning in ASD and TD.

For the deterministic condition, ANOVA revealed a main Block effect, *F*(4.4,311) = 13.1, *p* < .001, *ƞ*_*p*_^2^ = .16, reflecting a linear trend, *F*(1,70) = 28.0, *p* < .001, *ƞ*_*p*_^2^ = .29, suggesting learning. No main Group, *p* = .27, or Group × Block interaction, *p* = .097, effect was found, suggesting similar speed and learning in ASD and TD.

### Relation Between Sequence Learning and Social Impairment

#### Probabilistic Learning and Social Impairment

**Fig. 3:**
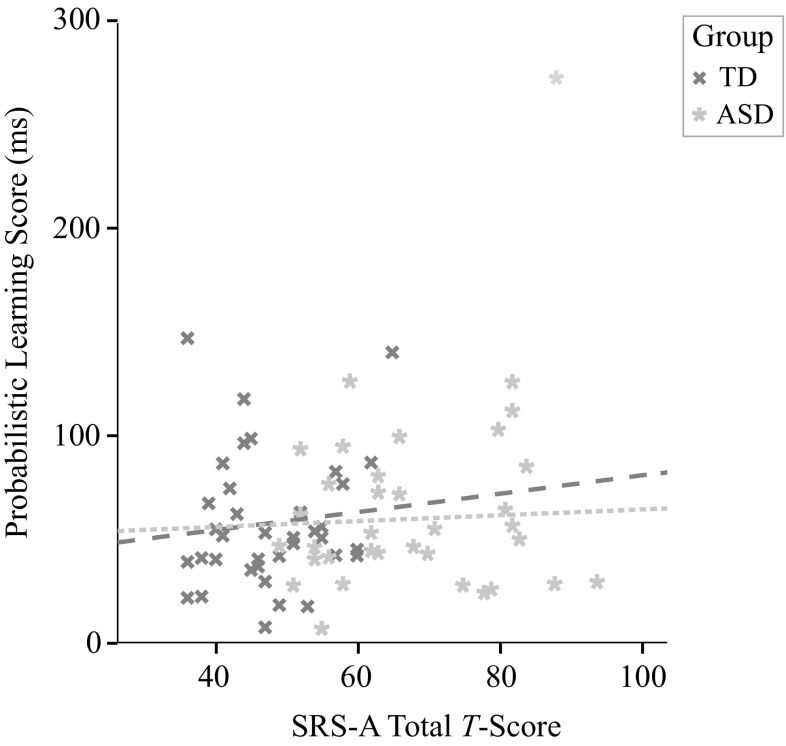
Correlation between learning in the probabilistic condition (i.e., mean RT deviant trials—mean RT standard trials) and SRS-A Total Score (*T*-score), with one outlier ASD participant in the upper right corner. Fit lines are shown after excluding this outlier participant

Figure 3 suggests no clear association between the probabilistic learning score and the SRS-A total score, and one outlier ASD participant. After excluding this participant (Cook’s distance = 1.035), indeed no correlation across groups (i.e., TD and ASD collapsed) was present, *r*(69) = .077, *p* = .53. No correlations were found within groups either, TD: *r*(35) = .11, *p* = .53; ASD: *r*(32) = .057, *p* = .75, after excluding the same outlier participant (Cook’s distance = 1.01).

#### Deterministic Learning and Social Impairment

**Fig. 4:**
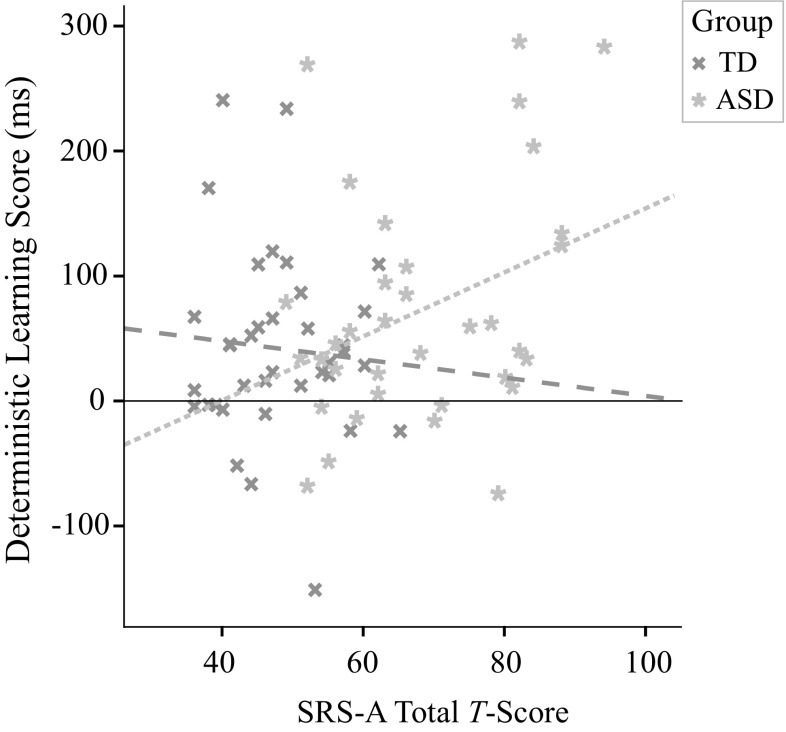
Correlation between learning in the deterministic condition (i.e., mean RT last block—mean RT first block) and SRS-A Total Score (*T*-score)

Figure 4 suggests a positive correlation between the deterministic learning score and the SRS-A total score, which was statistically confirmed, *r*(70) = .27, *p* = .023 (across groups). Within groups, a significant correlation was found for ASD, *r*(33) = .35, *p* = .041, but not for TD, *r*(35) = −.078, *p* = .65.

#### Differential Analyses on Age and IQ

Controlling for age and IQ did not change the general pattern of findings described above. That is, no correlations were found between probabilistic learning and the SRS-A total score, neither across groups or within groups. A positive correlation between deterministic learning and the SRS-A total score was found across group, and within-group confirmed for ASD but not for TD (see Supplementary Materials 2).

## Discussion

The aim of the current paper was to investigate the association between sequence learning on the SRT task and levels of social impairment related to ASD in autistic and non-autistic individuals. We used an SRT task with a probabilistic condition, designed to evoke implicit learning, and a deterministic learning condition that would allow for successful performance using explicit strategies. Overall performance on both conditions of the SRT task was similar in autistic and non-autistic individuals, in line with conclusions of previous meta-analyses and reviews on the topic (Foti et al. 2015; Obeid et al. 2016; Zwart et al. 2017a). An in-depth discussion of the current SRT task findings in ASD and TD falls beyond the scope of the current manuscript, but can be found in Zwart et al. 2017b.

The current findings suggest no association between probabilistic learning and social impairments as measured with the SRS-A. This was true for both the ASD and the TD group. This finding, especially in TD, does not corroborate the general idea that the type of implicit learning needed to successfully complete an SRT task is also involved in the development of social communication skills. A possible explanation is that although social communication skills depends upon the detection of temporal and spatial sequences of facial, gestural and vocal cues (e.g., Lieberman 2000), these sequences differ from the sequences used in an SRT task in terms of complexity and probability. For example, successful social communication skills includes understanding the other person, which requires probabilistically associating the facial, gestural and vocal sequences to internal emotional states. These sequences can be much longer and can occur with a much lower probability than the eight element sequences used in the current SRT task.

Perhaps even more surprising is the positive correlation found for deterministic learning and social impairments. Analyses of the ASD and TD groups separately revealed that this association was only significantly present in ASD, and did not seem to be driven by age or IQ. It seems that autistic individuals who are better at deterministic learning also experience more difficulties in social situations. Although this may sound counterintuitive, it could be explained by the idea of an overactive or compensatory explicit learning system in ASD (Klinger et al. 2007; Ullman and Pullman 2015). It has been found that explicit learning can be helpful in one situation, but detrimental in more complex situations (e.g., Howard and Howard 2001). It may be that autistic individuals who have a stronger developed explicit learning system benefit from this in a deterministic SRT task, but are hindered during learning from complex social situations. For example, learning the art of small talk in an explicit fashion would be extremely difficult and load heavily on cognitive resources, as in any such interaction the possible number of verbal and non-verbal cue sequences would be practically impossible to consciously predict and infer.

The finding of a positive correlation between deterministic learning and social impairments in autistic individuals is not in line with an exploratory analysis of a previous study reporting no such correlation in ASD (Travers et al. 2010). In this study, learning was measured as the difference between blocks of a deterministic sequence and one random block of trials near the end of the experiment. Important differences between the two studies that could explain the inconsistent findings, are the different SRS and learning measures. Travers et al. (2010) used raw scores from the parental version of the SRS for their group of 14- to 25-year-olds, whereas we used *T*-scores from the self-report version for adults. The parental version of the SRS (including norms to calculate *T*-scores) used by Travers et al. (2010) is developed for children and adolescents up to 18 years old, and was therefore not suitable for their older adult participants. Hence it could be argued that our measure of social impairments in adults is more accurate. However, our deterministic learning score included an RT difference over time, and could therefore reflect a general improvement in motor speed rather than sequence-specific learning (for references on motor (learning) impairments in ASD, see Dziuk et al. 2007; Fournier et al. 2010). Travers et al. (2010) did control for general motor speed by comparing sequenced trials with a block of random trials, and thereby measured sequence-specific learning more accurately than we did. Given a lack of such control in our study, one cannot exclude the possibility that the association with the SRS-A score was entirely driven by this general motor learning. However, this possibility is quite implausible given the direction of the observed association: the larger the RT decrease the *more* social impairments. Still, future research should include a random block at the end of the experiment and incorporate the performance on this block in the measure of sequence learning.

Taken together, it seems that social impairments in autism are related to the tendency to use explicit strategies during sequence learning (as presumably evoked by the deterministic condition) rather than to impairments in implicit learning (as presumably evoked by the probabilistic condition). However, the lack of correlations with probabilistic learning on the SRT task in both groups could be interpreted as a failure of the task to measure the implicit learning abilities we use to extract the complex statistical properties of our daily life environment, perhaps due to the task’s simplicity. For example, Lieberman (2000) describes how non-verbal communication requires a complex, and probabilistic sequencing of cues, such as hand gestures and facial expressions. The ecological validity of the SRT task could potentially be improved by increasing task complexity, for example, by decreasing the probability of the stimuli and by increasing sequence length.

Previous studies on the association between learning on the SRT task and everyday skills are mixed. Some studies have reported that learning on the SRT task predicts grammar abilities (Misyak et al. 2010; Lum et al. 2012), although one large study reported no association between learning on an SRT task and reading ability (Waber et al. 2003). The use of individual learning scores has been criticized by researchers using a different, statistical learning paradigm, mainly because quite a few individuals do not show learning on this task (Siegelman et al. 2017). This may also be a concern to learning on the SRT task, which is closely related to learning on the statistical learning paradigms (see Perruchet and Pacton 2006). Indeed, some of the deterministic learning scores in the current study were negative, i.e., the participants became *slower* over time, perhaps due to fatigue. It is important to develop a good derivative for individual learning on the SRT task, perhaps in which RT gains over all blocks are included, rather than the difference between the first and last block, as we decided a-priori. Several suggestions to develop a proper task and measure for individual sequence learning abilities has been made by Siegelman et al. (2017), including the use of trials with different probabilities, i.e., varying the difficulty level, which would increase the sensitivity to individual learning abilities.

Limitations of the current study include a relatively low sample size for correlational analyses, particularly the within-group analyses, and potentially the fixed order of task conditions in which the probabilistic condition was always followed by the deterministic condition. Because deterministic sequence learning is more likely to lead to awareness than probabilistic learning (e.g., Cleeremans and Jiménez 1998; Norman et al. 2007), starting with the deterministic condition could have triggered an active search for prompts in the following probabilistic condition, harming its implicit nature. Although the current design suits the current study aims best, we cannot rule out that learning the first, probabilistic part, has influenced (e.g., enhanced) learning on the second, deterministic part. The effect of task order could be investigated by counterbalancing the conditions in a larger study. Additionally, future studies could monitor learning over time rather than investigating only the current moment, as several clinical studies suggest deficits in consolidation of learning rather than in initial learning (e.g., Hedenius et al. 2011; Nemeth et al. 2013). And in everyday life, a skill is only useful if it can be used at a later point in time.

In conclusion, the current study suggests that better performance on a deterministic SRT task is associated with higher levels of social impairments in autistic participants as measured by the SRS-A. Probabilistic sequence learning does not seem to be related to social impairments. These findings suggest that caution should be taken in translating findings from traditional SRT studies to learning in everyday life, and call for further investigating the ecological validity of the SRT task. Furthermore, it would be interesting to replicate these findings using other measures of social impairment.

## Electronic supplementary material

Below is the link to the electronic supplementary material.

Supplementary material 1 (DOCX 13 KB)
