# Supplementary material for: The Association Between Sequence Learning on the Serial Reaction Time Task and Social Impairments in Autism
Source: J Autism Dev Disord. 2018 Mar 9;48(8):2692–700. doi: 10.1007/s10803-018-3529-6 (PMC6061016; doi:10.1007/s10803-018-3529-6)
Supplement: Supplementary file 1 — Supplementary material 1 (DOCX 13 KB) [file 10803_2018_3529_MOESM1_ESM.docx]

# Supplementary Materials 1

Two ANOVA’s with Study (1,2) as between-subject factor were conducted in order to confirm equivalence between the datasets from the two studies, and hence supporting collapsing the data across studies.

In the probabilistic condition, a Study (1,2) × Group (ASD,TD) × Trial Type (Standard,Deviant) × Block (9) ANOVA revealed no interaction effect of Study with Trial Type (the effect of interest): *F*(1,68)=.53, *p*=.47, *ƞ_p_*²=.008. Furthermore, no statistically significant Study × Trial Type × Block: *F*(4.38, 298)=.74, *p*=.58, *ƞ_p_*²=.011, or Study × Group × Trial Type × Block: *F*(4.38, 298)=2.28, *p*=.055, *ƞ_p_*²=.032, interaction effects were found.

In the deterministic condition with a Study (1,2) × Group (ASD,TD) × Block (9) ANOVA revealed no interaction of Study with Block (the effect of interest): *F*(4.52,307)=.21, *p*=.95, *ƞ_p_*²=.003. Furthermore, no Study × Group × Block interaction effect was found: *F*(4.52,307)=1.31, *p*=.26, *ƞ_p_*²=.019.

# Supplementary Materials 2

Table 2. Correlations between learning scores and SRS-A total score after controlling for age and IQ respectively

| Correlation SRS-A with | Group | Controlling for age | Controlling for IQ |
| --- | --- | --- | --- |
| Probabilistic learning | Across groups | *r*(68)=.092, *p*=.45 | *r*(68)=.051, *p*=.67 |
|  | ASD | *r*(31)=.059, *p*=.74 | *r*(31)=.002, *p*=.99 |
|  | TD | *r*(34)=.086, *p*=.62 | *r*(34)=.12, *p*=.47 |
| Deterministic learning | Across groups | *r*(69)=.24, *p*=.041* | *r*(69)=.28, *p*=.017* |
|  | ASD | *r*(32)=.34, *p*=.049* | *r*(32)=.40, *p*=.021* |
|  | TD | *r*(34)=-.045, *p*=.79 | *r*(34)=-.088, *p*=.61 |

*** p*-value < .05*
